# Validity of Verbal Autopsy Procedures for Determining Malaria Deaths in Different Epidemiological Settings in Uganda

**DOI:** 10.1371/journal.pone.0026892

**Published:** 2011-10-27

**Authors:** Arthur Mpimbaza, Scott Filler, Agaba Katureebe, Steven O. Kinara, Emmanuel Nzabandora, Linda Quick, Amy Ratcliffe, Fred Wabwire-Mangen, Daniel Chandramohan, Sarah G. Staedke

**Affiliations:** 1 Uganda Malaria Surveillance Project Kampala, Kampala, Uganda; 2 School of Medicine, College of Health Sciences, Child Health and Development Centre, Makerere University, Kampala, Uganda; 3 Centers for Disease Control and Prevention, Atlanta, Georgia, United States of America; 4 Department of Epidemiology and Biostatics, School of Public Health, College of Health Sciences, Makerere University, Kampala, Uganda; 5 London School of Hygiene & Tropical Medicine, London, United Kingdom; Aga Khan University, Pakistan

## Abstract

**Background:**

Verbal autopsy (VA) procedures can be used to estimate cause of death in settings with inadequate vital registries. However, the sensitivity of VA for determining malaria-specific mortality may be low, and may vary with transmission intensity. We assessed the diagnostic accuracy of VA procedures as compared to hospital medical records for determining cause of death in children under five in three different malaria transmission settings in Uganda, including Tororo (high), Kampala (medium), and Kisoro (low).

**Methods and Findings:**

Caretakers of children who died in participating hospitals were interviewed using a standardized World Health Organization questionnaire. Medical records from the child's hospitalization were also reviewed. Causes of death based on the VA questionnaires and the medical records were assigned independently by physician reviewers and then compared. A total of 719 cases were included in the final analysis, 67 in Tororo, 600 in Kampala, and 52 in Kisoro. Malaria was classified as the underlying or contributory cause of death by review of medical records in 33 deaths in Tororo, 60 in Kampala, and 0 in Kisoro. The sensitivity of VA procedures for determining malaria deaths in Tororo was 61% (95% CI 44–78%) and 50% in Kampala (95% CI 37–63%). Specificity for determining malaria deaths in Tororo and Kampala was high (>88%), but positive predictive value varied widely, from 83% in Tororo to 34% in Kampala (difference 49%, 95% CI 31–67, p<0.001). The difference between the cause-specific mortality fraction for malaria as determined by VA procedures and medical records was −11% in Tororo, +5% in Kampala, and +14% in Kisoro.

**Conclusions:**

Our results suggest that these VA methods have an acceptable level of diagnostic accuracy for determining malaria deaths at the population level in high and medium transmission areas, but not in low transmission areas.

## Introduction

Despite recent evidence that the burden of malaria is decreasing in many endemic areas [1], malaria remains one of the most serious global health problems [2]. In 2005, the United States launched the President's Malaria Initiative (PMI) to help control malaria in Africa (http://www.fightingmalaria.gov/). The primary goal of the PMI is to reduce malaria-related deaths by 50% in fifteen target countries in Africa, including Uganda. However, providing accurate estimates of malaria-specific mortality in resource-poor settings remains a challenge [3]. Many malaria deaths occur at home and are not documented [4]. Even if cases reach the formal health system, weak vital registration and health management information systems, and lack of good quality data on determinants of death, limit data collection and mortality estimates [4]–[6].

Verbal autopsy (VA) is an indirect method of determining cause of death based on an interview with the caretakers of a deceased individual, which has been widely used to collect information on cause-specific mortality where medical information on deaths is incomplete [7]. Information about specific signs and symptoms, and circumstances preceding the terminal event, are used to ascertain the most likely cause or causes of death [8]. Diagnoses are assigned and coded, and are then used to estimate cause-specific mortality fractions [9]. However, there is no widely accepted method of interpretation of the data, and VA procedures used at different sites vary substantially [8], [10], [11]. Variation in VA procedures limits comparability of data collected in different settings [12].

Studies evaluating the validity of VA procedures in African children indicate that sensitivity of verbal autopsy for determining malaria-specific mortality may be lower than for other diseases, and may vary with transmission intensity [13]–[17]. The positive predictive value (PPV) of VA procedures also varies with malaria prevalence, which may influence the performance of VA in different epidemiological settings [18]. Indeed, there is a theoretical concern that the imperfect sensitivity and specificity of VA could lead to an underestimation of the impact of the control efforts on malaria-specific mortality, even with a truly successful program. Efforts at improving and standardizing VA procedures are underway [19], but there is urgent need for data on the accuracy of VA methods in different settings.

We conducted a prospective study to evaluate the validity of VA procedures, in three different epidemiological settings in Uganda, using a questionnaire developed by the World Health Organization. The primary objective was to calculate the sensitivity, specificity, predictive values, and accuracy of cause-specific mortality fractions (CSMF) of VA procedures for attributing deaths to malaria as compared to review of medical records as the “gold standard” and to compare results between the different epidemiological settings.

## Methods

### Ethics

The study was approved by the Ugandan National Council for Science and Technology, the Centers for Disease Control and Prevention, and the ethics committees of Makerere University Faculty of Medicine, and the London School of Hygiene and Tropical Medicine. Permissions were obtained from district officials and medical superintendents at each hospital.

### Study site

The trial was conducted between June 2008 and September 2009 in five hospitals in three districts with different epidemiology including Tororo (rural, high malaria transmission), Kampala (urban, medium transmission), and Kisoro (rural, low transmission). Tororo and Kisoro districts are served by two hospitals; both were included from each district. MulagoHospital was chosen as the single hospital from Kampala because it is the main public hospital and serves all socio-economic groups. The estimated entomological inoculation rate is 562 in Tororo [20], 8 in Kampala (H. Hopkins, unpublished data), and<1 in Kisoro [21].

### Training

Prior to the study, staff in participating hospitals were trained, in accordance with standard hospital procedures, to help improve the quality of medical record keeping. Training emphasized the importance of documenting the full physical address and relevant history and physical examination. The importance of obtaining a blood smear for malaria parasites in all children who present with a history of fever or a documented fever was also emphasized.

### Participants

Study personnel monitored hospital admissions and deaths of children in participating hospitals. If a death was identified, the medical record of the deceased child was reviewed for the initial screening criteria including: 1) age <5 years, 2) residence within 60 km of the participating hospital, and 3) adequacy of the medical record (based on legibility and documentation of the physical address and presenting signs and symptoms). If the initial criteria were met, the household was scheduled for a visit approximately one month after the child's death. Study personnel located households based on the physical address with the help of local leaders. If a household could not be located within three months, the case was excluded. When a household was located, study personnel were introduced by the local leaders and condolence parcels containing sugar, salt, and soap were presented in accordance with local custom. Written informed consent was sought from the parent or guardian of the deceased child. Caretakers of deceased children who fulfilled the entry criteria were enrolled, and asked to identify an appropriate respondent(s) for the interview, defined as a person who was able and willing to provide an accurate account of the circumstances leading to the child's death. Verbal consent from the respondents was obtained prior to the interviews using an information sheet. Medical records from the child's hospitalization were acquired after written consent was obtained.

### Interview procedures

Interviews were conducted by study personnel in the local languages using a standardized verbal autopsy (VA) questionnaire developed by the World Health Organization [22]. Study personnel were trained in administration of the questionnaire and approaches to manage sensitive interviews. If a respondent became emotional during an interview and was unable to continue, the interview was delayed or rescheduled. Respondents were not allowed to refer to available records from the hospitalization, such as medical records or death certificates, but were asked to rely on their memory of the events during the interview.

### Classification of cause(s) of death

Two causes of death for each deceased child were derived; one from the hospital medical records and a second from the completed VA questionnaire. Different sets of physicians independently reviewed the medical records and VA questionnaires to determine cause(s) of death. Physicians were not blinded to the study sites. Cause(s) of death were determined based on the physician's clinical judgment and were classified as immediate, underlying, or contributory causes and coded according to the International Standard of Classification of Diseases (ICD-10) guidelines [22]. Two independent physicians classified cause(s) of death for each case, and a third physician resolved discrepant results. The cause(s) of death determined by the third physician were taken as final. Physician reviewers were medical officers, with experience in general pediatric care, and were trained in certification of death following the ICD-10 guidelines.

### Primary objective and statistical analysis

The primary objective of the study was to calculate the sensitivity, specificity, predictive values, and accuracy of CSMFs of VA procedures for attributing deaths to malaria (in which malaria was classified as either the underlying or contributory cause of death) as compared to review of medical records as the “gold standard” in low/medium/high malaria transmission areas, and to compare the PPV of malaria deaths identified through VA between the different sites with variable malaria transmission. Comparisons were also made using two restricted definitions of malaria deaths: (1) considering all cases in which malaria was classified as the underlying cause of death only, and (2) considering only cases in which a blood smear result was documented, in which malaria deaths (classified as the underlying cause of death) were confirmed by a positive blood smear. For evaluation of all-cause mortality, and misclassification of malaria deaths, the restricted definition of malaria death (classified as the underlying cause of death without blood smear confirmation) was used.

### Sample size calculations

We tested the alternative hypothesis that a significant difference in PPV could be detected in sites with variable malaria transmission, estimating that the PPV would be 90% in high transmission, 70% in medium transmission and 50% in low transmission areas [7], [13]–[15], [23]. To detect at least a 15% difference between the sites, we estimated that 180 deaths would be needed in each group to obtain a statistical power of 80% with 95% significance. To capture 180 deaths attributable to malaria as determined by verbal autopsy procedures, we estimated that we would need to recruit approximately 600 children per site. Due to limited numbers of deaths in Tororo and Kisoro, this sample size was attained only in Kampala.

## Results

### Enrollment

**Figure 1:**
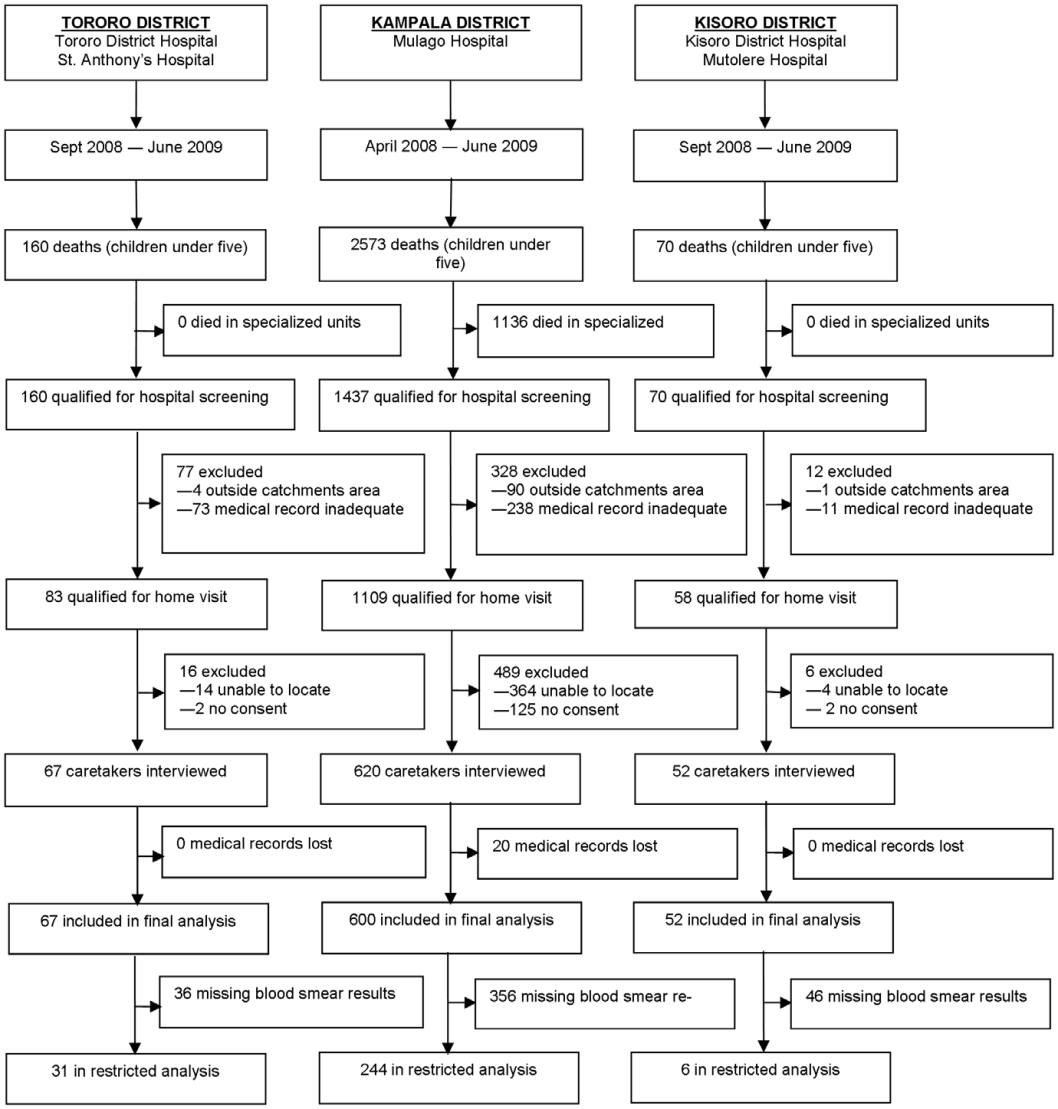
Study profile.

In Tororo, 160 deaths in children under five were recorded between September 2008 and June 2009; of these, 93 (58%) cases were excluded (Figure 1). All 67 cases enrolled were included in the final analysis, but only 31 (46%) had a blood smear result recorded and were included in the restricted analysis. In Kampala, 2573 deaths were recorded between April 2008 and June 2009; of these, 1953 (76%) cases were excluded, and 620 cases were enrolled. Of these cases, 600 were included in the final analysis; 244 (41%) had a blood smear result recorded and were included in the restricted analysis. In Kisoro, 70 deaths in children under five were recorded between September 2008 and June 2009; of these, 18 (26%) cases were excluded. All 52 cases enrolled in Kisoro were included in the final analysis; 6 (12%) had a blood smear result recorded and were included in the restricted analysis.

### Accuracy of VA procedures: Sensitivity, specificity, and positive predictive value

The sensitivity of VA procedures compared to medical records for determining malaria deaths, when malaria was classified as the underlying or contributory cause of death, ranged from 50% in Kampala to 61% in Tororo (Table 1). In Kisoro, although 6 malaria deaths were classified by VA procedures, none were classified by medical records. Specificity in both Tororo and Kampala was high (>88%), but PPV varied widely, from 83% in Tororo to 34% in Kampala (difference 49%, 95% confidence interval [CI] 31–67, p<0.001). When malaria deaths were limited to those in which malaria was classified as the underlying cause of death, the sensitivity, specificity and PPV of VA procedures changed little. When the analysis was restricted to cases with a blood smear result, and malaria deaths (classified as the underlying cause of death) were confirmed by a positive blood smear, results were also similar; sensitivity increased in Tororo to 71%, but remained 51% in Kampala; specificity dropped slightly (79% in Tororo, 84% in Kampala), and PPV remained stable in both sites.

**Table 1:** Accuracy of verbal autopsy procedures for determining malaria deaths as compared to medical records at three sites.

	Total deaths attributable to malaria		Sensitivity(%, 95% CI*)	Specificity(%, 95% CI)	PPV†(%, 95% CI)	CSMF_VA_ ‡(%, 95% CI)	CSMF_MR_ §(%, 95% CI)	CSMF_VA_– CSMF_MR_(%, 95% CI)
	Verbal autopsy	Verbal autopsy						
**All cases; malaria classified as the underlying or contributory cause of death**								
Tororo (N = 67)	24	33	61 (44, 78)	88 (77, 99)	83 (68, 98)	36 (25, 47)	49 (37, 61)	−13 (−29, 4)
Kampala (N = 600)	88	60	50 (37, 63)	89 (86, 92)	34 (24, 44)	15 (12, 18)	10 (8, 12)	5 (1, 9)
Kisoro (N = 52)	6	0	0	0	0	12 (3, 21)	0	12 (3, 21)
**All cases; malaria classified as the underlying cause of death**								
Tororo (N = 67)	24	32	63 (46, 80)	89 (79, 99)	83 (68, 98)	36 (25, 47)	48 (36, 60)	−12 (−28, 5)
Kampala (N = 600)	85	51	57 (43, 71)	90 (87, 93)	34 (24, 44)	14 (11, 17)	9 (7, 11)	5 (1, 9)
Kisoro (N = 52)	6	0	0	0	0	12 (3, 21)	0	12 (3, 21)
**Restricted to in cases with a blood smear result; malaria classified as the underlying cause of death confirmed by a positive blood smear**								
Tororo (N = 31)	15	17	71 (49, 93)	79 (58, 100)	80 (60, 100)	48 (30, 66)	55 (37, 73)	−7 (−32, 18)
Kampala (N = 244)	52	37	51 (35, 67)	84 (79, 89)	37 (24, 50)	21 (16, 26)	15 (10, 19)	6 (−1, 13)
Kisoro (N = 6)	0	0	0	0	0	0	0	0

*CI  =  Confidence interval; † PPV  =  Positive predictive value.

‡ CSMF_VA_  =  Cause-specific mortality fraction as determined by verbal autopsy procedures.

§ CSMF_ MR_  =  Cause-specific mortality fraction as determined from medical records.

### Accuracy of VA procedures: Cause-specific mortality fraction

Considering deaths in which malaria was classified as the underlying or contributory cause of death, the CSMF for malaria determined by VA procedures in Tororo was lower than that determined by medical records (Table 1), indicating that VA procedures underestimated malaria mortality (13% difference), although this was not statistically significant (p = 0.13). In Kampala and Kisoro, the opposite was found; VA procedures significantly overestimated malaria deaths (Kampala: 5% difference, p = 0.009; Kisoro: 12% difference, p = 0.01). Results were similar when malaria deaths were limited to the underlying cause of death. In the analysis restricted to cases confirmed by a positive blood smear, the differences were smaller, but the directions remained the same. VA procedures underestimated malaria deaths in Tororo (7% difference) and overestimated malaria deaths in Kampala (6% difference), although these differences were not statistically significant. In Kisoro, no malaria deaths determined by VA procedures were confirmed by a positive blood smear.

### Accuracy of VA procedures: All-cause mortality

**Figure 2:**
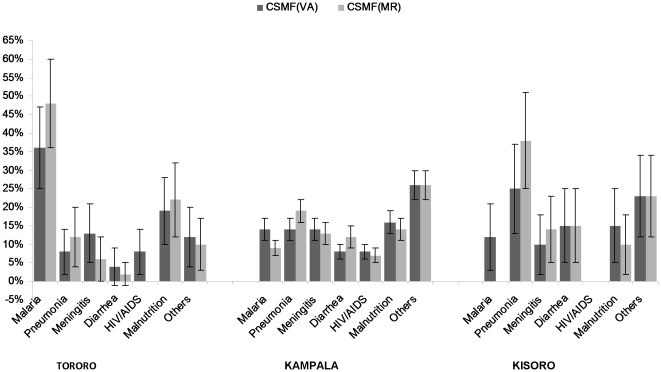
Cause-specific mortality fraction as established by verbal autopsy and medical records, by site.

To evaluate the accuracy of VA procedures for classifying non-malarial deaths, we determined the sensitivity and specificity of VA for other leading causes of death at each site. In Tororo, the leading causes of death were malaria and malnutrition; in Kampala, the majority of deaths were classified as “other” which included neonatal sepsis (26%), congenital malformations (14%), other neonatal causes of death (12%), tuberculosis (9%), septicemia (7%), anemia (7%), burns (6%) and others (19%); pneumonia was also a common cause of death (Table 2, Figure 2). In Kisoro, pneumonia was the leading cause of death. The accuracy of VA procedures for determining leading causes of death varied between sites. Specificity was generally high for all diseases at all sites, but sensitivity and PPV varied widely. For pneumonia, sensitivity and PPV were lowest in Tororo and highest in Kisoro. Sensitivity of VA procedures for determining meningitis deaths was high in Tororo but much lower in Kampala and Kisoro, and PPV ranged from 33% to 60%. Death due to diarrhea was uncommon in Tororo, but occurred more frequently in Kampala and Kisoro; sensitivity and PPV of VA procedures for diarrheal deaths was low in Kampala, but higher in Kisoro. For malnutrition, sensitivity was marginal at all sites, and PPV was particularly low in Kisoro (25%). The CSMFs determined by VA procedures and medical records generally differed by less than 10% for all diseases. The greatest difference was seen for pneumonia in Kisoro; VA procedures underestimated pneumonia deaths by 13%.

**Table 2:** Accuracy of verbal autopsy procedures for determining cause of death as compared to medical records at three sites.

Cause of death	Verbal autopsy	Medical records	Sensitivity(%, 95% CI)	Specificity(%, 95% CI)	PPV†(%, 95% CI)	CSMF_VA_ ‡(%, 95% CI)	CSMF_MR_ §(%, 95% CI)	CSMF_VA_– CSMF_MR_(%, 95% CI)
**Tororo District (N = 67)**								
Malaria*	24	32	63 (46, 80)	89 (79, 99)	83 (68, 98)	36 (25, 47)	48 (36, 60)	−12 (−28, 5)
Pneumonia	5	8	25 (−5, 55)	95 (89, 100)	40 (−3, 83)	8 (2, 14)	12 (4, 20)	−4 (−14, 6)
Meningitis	9	4	75 (32, 117)	90 (83, 97)	33 (2, 64)	13 (5, 21)	6 (0, 12)	7 (−3, 17)
Diarrhoeal illnesses	3	1	0	95 (90, 100)	0	4 (−1, 9)	2 (−1, 5)	2 (−4, 8)
HIV/AIDS	5	0	0	0	0	8 (2, 14)	0	8 (2, 14)
Malnutrition	13	15	53 (28, 78)	90 (82, 98)	62 (36, 88)	19 (10, 28)	22 (12, 32)	−3 (−17, 11)
Others	8	7	29 (−5, 63)	90 (82, 98)	25 (−5, 55)	12 (4, 20)	10 (3, 17)	2 (−9, 13)
**Kampala District (N = 600)**								
Malaria*	85	51	57 (43, 71)	90 (87, 93)	34 (24, 44)	14 (11, 17)	9 (7, 11)	5 (1, 9)
Pneumonia	85	111	39 (30, 48)	91 (88, 94)	51 (40, 62)	14 (11, 17)	19 (16, 22)	−5 (−9, −1)
Meningitis	86	78	49 (38, 60)	91 (86, 93)	44 (34, 54)	14 (11, 17)	13 (10, 16)	1 (−3, 5)
Diarrhoeal illnesses	46	74	30 (20, 40)	95 (93, 97)	48 (34, 62)	8 (6, 10)	12 (9, 15)	−4 (−7, −1)
HIV/AIDS	49	44	61 (47, 75)	96 (94, 98)	55 (41, 69)	8 (6, 10)	7 (5, 9)	1 (−2, 4)
Malnutrition	95	85	58 (48, 68)	91 (89, 93)	52 (42, 62)	16 (13, 19)	14 (11, 17)	2 (−2, 6)
Others	154	157	59 (51, 67)	86 (83, 89)	60 (52, 68)	26 (22, 30)	26 (22, 30)	0 (−5, 5)
**Kisoro District (N = 52)**								
Malaria*	6	0	0	0	0	12 (3, 21)	0	12 (3, 21)
Pneumonia	13	20	55 (33, 77)	94 (86, 102)	85 (66, 104)	25 (13, 37)	38 (25, 51)	−13 (−30, 5)
Meningitis	5	7	43 (6, 80)	96 (90, 101)	60 (17, 102)	10 (2, 18)	14 (5, 23)	−4 (−16, 8)
Diarrhoeal illnesses	8	8	75 (45, 105)	95 (89, 101)	75 (45, 105)	15 (5, 25)	15 (5, 25)	0 (−14, 14)
HIV/AIDS	0	0	0	0	0	0	0	0
Malnutrition	8	5	40 (−3, 83)	87 (77, 97)	25 (−5, 55)	15 (5, 25)	10 (2, 18)	5 (−8, 18)
Others	12	12	83 (62, 104)	95 (88, 101)	83 (62, 104)	23 (12, 34)	23 (12, 34)	0 (−16, 16)

*Malaria classified as the underlying cause of death.

† PPV  =  Positive predictive value.

‡ CSMF_VA_  = Cause-specific mortality fraction as determined by verbal autopsy procedures.

§ CSMF_ MR_  = Cause-specific mortality fraction as determined from medical records.

### Misclassification of malaria deaths

In Tororo, five deaths due to other illnesses were inaccurately attributed to malaria (false positives), and 12 deaths due to malaria were inaccurately attributed to other illnesses (false negatives, Table 3). The false positive and false negative causes of death were fairly well-distributed across the alternative diagnoses. In Tororo, no pattern of misclassification was apparent. Similarly in Kampala, the 56 deaths due other illnesses that were inaccurately attributed to malaria (false positives) were well-distributed across the alternative diagnoses. Of the 21 deaths due to malaria that were inaccurately attributed to other illnesses (false negatives) in Kampala, one-half were attributed to meningitis. In Kisoro, of the 6 false positive cases, one-half were due to pneumonia. No false negative cases occurred in Kisoro.

**Table 3:** Misclassification of malaria deaths, as the underlying cause of death, by verbal autopsy procedures.

	Tororo District		Kampala District		Kisoro District	
	False positive* (N = 5)	False negative† (N = 12)	False positive* (N = 56)	False negative† (N = 21)	False positive* (N = 6)	False negative† (N = 0)
Pneumonia (%)	1 (25)	0	10 (18)	1 (5)	3 (50)	0
Meningitis (%)	1 (25)	2 (17)	12 (21)	11 (52)	2 (33)	0
Diarrhea (%)	0	3 (25)	14 (25)	1 (5)	1 (17)	0
Malnutrition (%)	1 (25)	4 (33)	6 (11)	3 (14)	0	0
HIV/AIDS (%)	0	2 (17)	1 (2)	1 (5)	0	0
Other (%)	1 (25)	1 (8)	13 (23)	4 (19)	0	0

*False positive: Cause of death determined by VA procedures was inaccurately classified as malaria. The values represent the number and proportion of diagnoses that were incorrectly classified as malaria.

† **False negative**: Cause of death determined by VA procedures was inaccurately classified as an illness other than malaria. The values represent the number and proportion of true malaria cases that were incorrectly classified as other illnesses.

## Discussion

In this study, we evaluated the validity of VA procedures, using a standardized World Health Organization questionnaire and physician review to classify cause of death, in three different epidemiological settings in Uganda. In Tororo and Kampala, the high and medium malaria transmission sites, we found that the sensitivity of VA procedures for determining malaria deaths was ≥50%, specificity was >88%, and the CSMF was within 13% of the true value. No malaria deaths occurred in Kisoro, the low transmission site, but VA procedures overestimated the CSMF attributable to malaria by 12%. Our results suggest that these VA methods for determining malaria deaths have an acceptable diagnostic accuracy for use at the population level in high and medium transmission areas. In low transmission areas, VA is unlikely to be useful for measuring the impact of interventions on malaria burden, or for detecting a change from low to very low transmission.

In this study, the sensitivity and specificity of VA procedures for determining malaria deaths were fairly high, particularly in Tororo. In prior validation studies, the sensitivity of VA procedures for determining malaria deaths in African children has ranged widely, from 45% to 86% [7], [13]–[17], [23]. Specificity is also variable (67% to 100%) but is typically much higher than sensitivity [7], [13]–[17], [23]. The low sensitivity of VA for determining malaria deaths in these studies likely results from the non-specific nature of malaria symptoms which overlap substantially with symptoms of other common causes of death, particularly pneumonia and meningitis. Differences in the underlying mix of such diseases in study populations may explain the variability in sensitivity results across sites. Malaria transmission intensity, host immunity in the population, and typical presentations of malaria illness, also impact on VA sensitivity and may also explain variability of results across sites. In high transmission areas, young children with severe malaria tend to present with severe anemia, while in lower transmission areas, severe malaria may present in older children as cerebral malaria. The high sensitivity we observed in Tororo may be attributed to greater numbers of children with severe anemia, which may be recognized by specific signs and symptoms [12]. We also found that the PPV of VA for malaria deaths was significantly higher in Tororo than in Kampala, which is consistent with the differences in malaria endemicity and prevalence between the sites.

The accuracy of VA for estimating the CSMF for malaria varied in our study, underestimating the proportion of deaths due to malaria in the high transmission site, and overestimating it in the medium and low transmission sites. Because of the variability in sensitivity and specificity of VA procedures, cause-specific mortality estimates obtained by VA are susceptible to bias due to inaccurate classification of causes of death, resulting in under- or over-estimation of the true CSMF [24]–[26]. This can have a substantial effect on the VA estimate of the proportion of deaths due to a specific cause within a population. Indeed, it is likely that the results of a recently published study that used VA procedures to estimate malaria-associated mortality in India; a low transmission setting, may have substantially over-estimated the true burden of malaria in India [27], [28]. Misclassification not only impacts estimates of CSMF in a given population but may also affect comparison of CSMF between two population groups, and changes in CSMF over time [7].

Our results also show how the accuracy of VA in determining the CSMF for malaria is dependent on the direction and magnitude of misclassification. In our study, misclassification errors occurred at all sites. In Kampala, one-half of the false negative cases (in which the cause of death determined by VA was inaccurately attributed to an illness other than malaria) were misclassified as meningitis. In Kisoro, one-half of the false positive cases (in which the cause of death determined by VA was inaccurately classified as malaria) were in fact due to pneumonia. However, no obvious patterns of misclassification were found in our study. It is possible to adjust for misclassification if the sensitivity and specificity of VA procedures is known [29]. However, it is unclear if test characteristics estimated from hospital-based validation studies can be applied to a community setting [7], [18]. The previously reported methods to adjust the VA estimates of malaria deaths for misclassification are unlikely to be useful in very low transmission settings [29]. Application of sensitivity and specificity of VA obtained in high/medium transmission settings to adjust the effect of misclassification is unlikely to give the true estimate of malaria deaths in very low transmission settings.

The most significant limitation of this study was the quality of the “gold standard”, including the in-patient care provided and documentation in the medical records. Validation studies typically compare the sensitivity and specificity of VA diagnoses against causes of death established by medical records as the gold standard [8]. Although we attempted to improve medical record keeping by training staff in all participating hospitals prior to beginning the study, the quality of medical records remained marginal, resulting in a large number of exclusions during screening. Poor documentation could be attributable to poor information provided by the caregiver, or poor history taking or recording on the part of the provider. However, it is unlikely that the exclusions due to inadequate medical records were related to the child's underlying disease state or would have affected the nature of the findings.

In addition, barriers to obtaining laboratory tests existed at most hospitals, limiting use of microscopy, as evidenced by the low numbers of cases with a blood smear result recorded. Despite these limitations, when the accuracy of VA procedures was compared to a more refined “gold standard”, in which malaria deaths (as the underlying cause of death) were confirmed by a positive blood smear, the results were similar, supporting the validity of our data.

Our study had several other significant limitations. The proportion of cases excluded during screening was high, which may have resulted in selection bias. In Tororo, most cases were excluded due to inadequate medical records, while in Kampala, inadequate documentation of physical addresses and difficulty tracing households contributed to the exclusions. In addition, we were unable to reach our sample size targets in Tororo and Kisoro due to low numbers of deaths, which limited our ability to assess for variations in the PPV of VA procedures for determining malaria deaths between the sites, particularly in Kisoro where no malaria deaths were captured, limiting attempts at determining the accuracy of VA procedures at this site. However, despite not reaching the sample size target in Tororo, the observed difference in PPV of VA procedures for determining malaria deaths between Tororo and Kampala was significant. In this study, we relied on physician review to classify cause of death. Use of standardized algorithms to derive cause of death from VA questionnaires has been advocated as a way to systematically assign diagnoses, and to save time [30]. However, the most widely used approach to deriving cause of death remains physician review [8]. The validity of physician review for interpreting VA data has been evaluated in several studies, and has been shown to be sensitive and specific for selected causes of death [9]. However, use of physician review has been criticized for its subjectivity and low reliability [9], [11], [31]. Use of algorithms to derive cause of death could increase reliability of VA procedures and allow for automation of the coding process. Two types of algorithms have been developed, expert and data-derived; however, the performance of both types of algorithms appear to be lower than physician review for determining cause of death in adults, and require further evaluation [30].

In summary, our results suggest that these VA procedures have a high diagnostic accuracy for determining malaria deaths at the population level in high and medium transmission areas, but not in low transmission areas. For other causes of death, VA provided moderate sensitivity and high specificity regardless of transmission level. As malaria transmission falls to very low levels, VA is likely to overestimate malaria-specific mortality and underestimate the impact of control interventions. Indeed, there is likely to be a “tipping point” in malaria transmission where VA procedures cease to be useful, but defining this threshold may not be possible due to methodological constraints. VA procedures appear to have a role in estimating malaria-specific mortality, but only in high and medium transmission areas; in low transmission areas, the utility of VA appears to be limited.
